# The application of machine learning in clinical microbiology and infectious diseases

**DOI:** 10.3389/fcimb.2025.1545646

**Published:** 2025-05-01

**Authors:** Cheng Xu, Ling-Yun Zhao, Cun-Si Ye, Ke-Chen Xu, Ke-Yang Xu

**Affiliations:** ^1^ Clinical Laboratory of Chun’an First People’s Hospital, Zhejiang Provincial People’s Hospital Chun’an Branch, Hangzhou Medical College Affiliated Chun’an Hospital, Hangzhou, Zhejiang, China; ^2^ Department of Medicine & Therapeutics, Prince of Wales Hospital, The Chinese University of Hong Kong, Hong Kong, Hong Kong SAR, China; ^3^ Department of Clinical Laboratory Medicine, Institution of Microbiology and Infectious Diseases, The First Affiliated Hospital, Hengyang Medical School, University of South China, Hengyang, Hunan, China; ^4^ School of Psychology, Zhejiang Normal University, Jinhua, China; ^5^ Key Laboratory of Intelligent Education Technology and Application of Zhejiang Province, Zhejiang Normal University, Jinhua, China; ^6^ Faculty of Chinese Medicine, and State Key Laboratory of Quality Research in Chinese Medicine, Macau University of Science and Technology, Macao SAR, China

**Keywords:** machine learning, artificial intelligence, clinical microbiology, infectious diseases, application

## Abstract

With the development of artificial intelligence(AI) in computer science and statistics, it has been further applied to the medical field. These applications include the management of infectious diseases, in which machine learning has created inroads in clinical microbiology, radiology, genomics, and the analysis of electronic health record data. Especially, the role of machine learning in microbiology has gradually become prominent, and it is used in etiological diagnosis, prediction of antibiotic resistance, association between human microbiome characteristics and complex host diseases, prognosis judgment, and prevention and control of infectious diseases. Machine learning in the field of microbiology mainly adopts supervised learning and unsupervised learning, involving algorithms from classification and regression to clustering and dimensionality reduction. This Review explains crucial concepts in machine learning for unfamiliar readers, describes machine learning’s current applications in clinical microbiology and infectious diseases, and summarizes important approaches clinicians must be aware of when evaluating research using machine learning.

## Introduction

In 1956, John McCarthy and colleagues founded the field of artificial intelligence at an artificial intelligence conference at Dartmouth College that spawned a new interdisciplinary field of study (Kaul et al., 2020). AI is a new technical science that studies and develops theories, methods, technologies, and application systems used to simulate, extend, and expand human intelligence. AI involves robotics, language recognition, image recognition, natural language processing, expert systems, machine learning, computer vision, etc. The application of AI in medicine has two main branches: virtual and physical. The virtual component is represented by machine earning that uses mathematical algorithms for improving learning through experience (Kaul et al., 2020). The second form of application includes physical objects, medical devices, and increasingly sophisticated robots taking part in the delivery of care (Cornet, 2013).

**Figure 1:**
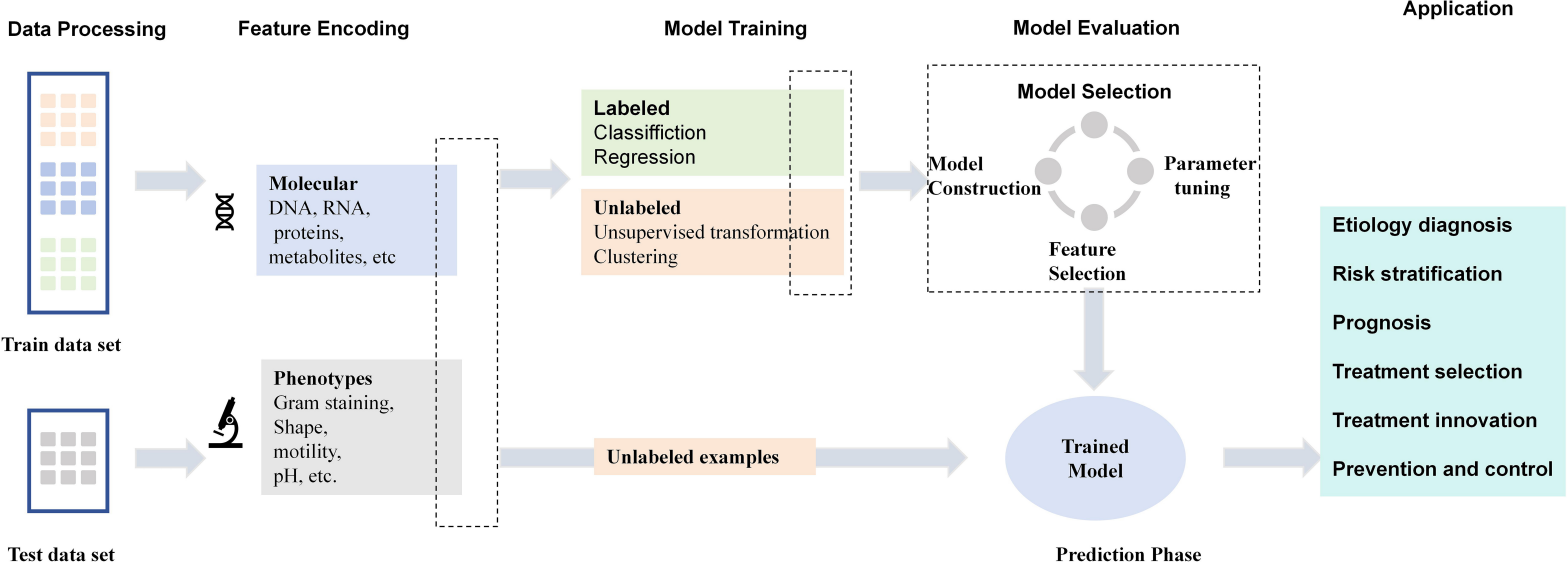
The development and application for example machine learning model. The building process of machine learning model is mainly from data processing, feature coding, model training, model evaluation and selection, and finally to test data prediction. The applications in clinical microbiology and infectious diseases are included.

Machine learning emerges at the intersection of statistics and computer science, where the convergence of the two disciplines is driven by the unique computational challenge of building statistical models from massive data sets (Deo, 2015). There are three types of machine learning algorithms: unsupervised learning, supervised learning, and reinforcement learning. From the perspective of medicine, machine learning’s substantial progress carries potential implications across the scope of practice, including drug research, disease diagnosis, risk stratification and prognosis, treatment planning, and advances in precision medicine approaches (Deo, 2015; Radakovich et al., 2020). Data from various omics sources such as genetics, proteomics, and metabolomics can be integrated to unravel the intricate workings of systems biology using predictive algorithms, such as the discovery of markers (Reel et al., 2021). These new biomarkers have the potential to help in accurate disease prediction, patient stratification, and delivery of precision medicine (Reel et al., 2021). In addition, details of the building process of machine learning can be seen in **Figure 1**, including data processing, feature encoding, model training and model evaluation

The applications in clinical microbiology and infectious diseases are quickly expanding, used in etiological diagnosis, prediction of antibiotic resistance, association between human microbiome characteristics and complex host diseases, prognosis judgment, and prevention and control of infectious diseases (Asnicar et al., 2024). In this Review, we want to help clinical staff grasp the important concepts and basic applications of machine learning ranging from their experiments to the critical assessments of the work. Firstly, we introduce supervised and unsupervised machine learning techniques, especially focusing on muti-omics data analysis. We also examine approaches for algorithms of machine learning, for example, dimensionality reduction is frequently used for exploratory microbiological investigations, and feature selection is key to identifying the most relevant aspects of the microbiological phenomenon. Secondly, we mainly summarized the application of machine learning in clinical microbiology and infectious diseases, from diagnosis, risk stratification, prognosis, treatment selection, and response prediction to infectious disease prevention and control, and multi-omics applications. Thirdly, we highlight the key elements of machine learning for clinical staff, including how to evaluate machine learning models and how to apply them to real-world scenarios that minimize potential bias.

## Supervised machine learning

Supervised machine learning, uses training sets of input/output pairs to build machine learning models designed to make accurate predictions about new data that have never been seen before. Supervised learning usually requires a certain amount of manpower to build the training set, mainly by labeling the corresponding data features such as the sequence of the gene or genome of the strain, or phenotypic information obtained by *in vitro* experiments on the strain. Labeled data usually defines the outcome of interest, for example, to train an algorithm for sepsis prediction, we use a dataset in which patients are already defined as having sepsis or not (Peiffer-Smadja et al., 2020). Besides, supervised learning improves outbreak detection of Salmonella and Campylobacter infections using routine surveillance data (Zacher and Czogiel, 2022), diagnoses the childhood febrile illness using a multi-class blood RNA molecular signature (Habgood-Coote et al., 2023) and inflammatory bowel diseases using gut microbiome (Manandhar et al., 2021), screens and types diabetes using gut microbiome metagenomic hypervariable features (Chavarria et al., 2025), etc. Supervised learning usually divides labeled data into training sets and verification sets, and unlabeled data into test sets. Common algorithms for supervised machine learning include Decision Trees(DT), Ordinary Least Squares Regression(OLS), Naive Bayesian classification(NB), Logistic Regression(LR), Support Vector Machine(SVM), Ensemble methods(EM), Random Forests linear regression(RF), Linear Discriminant Analysis (LD), k-nearest neighbor algorithm(k-NN), Multi-layer perceptron(MLP), Convolutional neural networks(CNN)and so on.

There are two main types of supervised machine learning problems, called classification and regression. When supervised learning uses categorical labels (for example, taxonomic labels) for the outcome variable, it is referred to as classification, whereas regression refers to the case in which the outcome variable is a numerical continuous variable (for example, the optimal pH for a bacterium to grow) (Asnicar et al., 2024). Supervised learning focuses on classification, which involves choosing among subgroups to best describe a new instance of data, and prediction, which involves estimating an unknown parameter (Deo, 2015). For example, machine learning techniques through RF and gradient boosting (GB) models can be successfully applied to predict malaria using patient information (Lee et al., 2021). The graph-based MLP and RF models effectively diagnosed influenza and hepatitis, respectively (Alqaissi et al., 2023). In addition, machine learning algorithms for taxonomic classification of 16S rRNA genes from isolate sequences or of 16S rRNA gene fragments from microbiome experiments have been developed for this task, including *k*-mer profiling and support vector machines (SVMs) (McHardy et al., 2007; Diaz et al., 2009; Gregor et al., 2016; Vervier et al., 2016). However, different algorithms have their own advantages and disadvantages. For example, the most important advantage of RF is that training can be highly parallelized, which has advantages for large-sample training speed in the era of big data (Hu and Szymczak, 2023). However, RF models tend to fall into overfitting with some noisy sample sets and features with more value division (Hu and Szymczak, 2023). The SVM algorithm is very effective in high-dimensional feature classification and regression problems, and it still has a good effect when the feature dimension is larger than the number of samples (Valkenborg et al., 2023). However, SVM algorithm is not suitable for big data and sensitive to missing data (Valkenborg et al., 2023). The main advantages of DT algorithm are that data preprocessing is relatively simple and missing data can be processed (Kingsford and Salzberg, 2008). However, DT algorithm is very easy to overfit, resulting in poor generalization ability, and is not suitable for complex relationships and characteristic samples (Kingsford and Salzberg, 2008). The KNN regression method, known for its nonparametric nature, is highly valued for its simplicity and its effectiveness in handling complex structured data, particularly in big data contexts (Srisuradetchai and Suksrikran, 2024). However, this method is susceptible to overfitting and fit discontinuity, which present significant challenges (Srisuradetchai and Suksrikran, 2024). The naive Bayes algorithm is stable for classification efficiency, missing data, and large data sets (Awaysheh et al., 2019). However, Bayesian algorithms are often influenced by prior probability models (Awaysheh et al., 2019). CNN has the high classification, and strong robustness and fault tolerance to noise nerve (Shan et al., 2021). However, CNN require a large number of parameters, such as the network topology, weights, and initial values of thresholds (Shan et al., 2021).

## Unsupervised machine learning

Unsupervised learning can solve various problems in pattern recognition based on training samples whose class is unknown (not labeled), such as finding subsets of patients with similar expression levels in a gene expression study (Altman and Krzywinski, 2017) or predicting mutation effects from gene sequence co-variation (Hopf et al., 2017). What’s more, the measurement of the gene expression time point of each cell in the isogenic bacterial cell population in liquid batch culture at different points should identify the cell growth stage and the cell group with similar gene expression could be divided by unsupervised learning algorithm to reflect the overview of the growth pattern (Asnicar et al., 2024). There are two types of unsupervised learning: unsupervised transformation and clustering algorithm. Unsupervised transformation of a data set is the creation of a new representation of the data that may be easier for humans or other machine learning algorithms to understand than the original representation of the data. The clustering algorithm divides the data into different groups by identifying similar items.

Dimensionality reduction, a common application of unsupervised transformation, is a new way of accepting a high-dimensional approach to data that contains many features, with fewer features to generalize its important properties. For example, dimensionality reduction could be applied to visualize single-cell data (Amouzgar et al., 2022; Becht et al., 2018; Jiang et al., 2023), investigate the diversity of Tuberculosis Spoligotypes (Senelle et al., 2022), deconvolute gut microbial community dynamics (Martino et al., 2021), distill complex evolutionary relationships in seasonal influenza and SARS-CoV-2 (Nanduri et al., 2024), characterize circulating innate lymphoid cell in inflammatory bowel disease (Mazzurana et al., 2021), treat for human immunodeficiency virus infection (Choi et al., 2023), etc. These techniques comprise both linear and non-linear transformations of the data, including principal component analysis (PCA) (Tsuyuzaki et al., 2020), Uniform Manifold Approximation and Projection (UMAP) (Becht et al., 2018) and t-distributed stochastic neighbor embedding (t-SNE) (Kobak and Berens, 2019). PCA is a technique for reducing the dimensionality of such datasets, increasing interpretability but at the same time minimizing information loss (Jolliffe and Cadima, 2016). Uniform Manifold Approximation and Projection (UMAP) is an alternative method that can reduce the dimensionality of beta diversity distance matrices, for example, UMAP can reveal composite patterns and resolve visualization artifacts in microbiome data (Armstrong et al., 2021). Common data analysis pipelines include a dimensionality reduction step for visualizing the data in two dimensions, most frequently performed using t-distributed stochastic neighbor embedding, for example, in single-cell transcriptomics (t-SNE) (Kobak and Berens, 2019). Dimensionality reduction can improve computing efficiency, reduce storage space, remove redundant features, speed up follow-up processing, and promote visualization. The main disadvantages of dimensionality reduction in machine learning algorithms include loss of information, decreased interpretability of and higher technical barriers.

Clustering methods are used to predict groupings of similar data points in a dataset and are usually based on some measure of similarity between data points (Greener et al., 2022). For example, clustering could be applied to analyze gene sequence (Ali et al., 2022; He et al., 2023), visualize the sequence-structure-function relationship of protein networks (Mai et al., 2016; Mirdita et al., 2017; Yeung et al., 2023), densely sample human gut microbiome time series (Benincà et al., 2023), categorize countries into homogeneous subgroups based on the joint patterns of HIV/AIDS and TB mortality rates (Mobaderi et al., 2025), detect infectious disease transmission outbreaks from sequence variation (McCloskey and Poon, 2017), etc. The clustering algorithm mainly includes K-means clustering, agglomerative clustering, and density-based spatial clustering of applications with noise (DBSCAN). K-means clustering can identify diverse clinical phenotypes in COVID-19 patients (Garcia-Vidal et al., 2024) and estimate bacterial community composition (Koslicki et al., 2015). The agglomerative clustering algorithm can reveal distinct community types of the bacterial populations (Bezek et al., 2020; Ghosh et al., 2022). Clustering algorithm has the advantages of flexibility and simplicity, which can handle noise and no need to specify the number of clusters in advance. However, the clustering algorithm has high computational complexity and sensitive parameters, which needs to determine the number of clusters in advance.

## Other machine learning paradigms

Semi-supervised learning trains a model using not only labeled data generally available in small amount, but also using unlabeled data often available in large amount (Mourad, 2023). For example, semi-supervised learning could be applied to predict virus-receptor interactions (Yan et al., 2019), facilitate antibiotic stewardship for urinary tract infections (de Vries et al., 2022), predict *Lactobacillus delbrueckii* subsp. bulgaricus-Streptococcus thermophilus interactions (Yang et al., 2025), segment the medical image (Tang et al., 2023), etc. Key technical approaches include: 1) Consistency regularization (e.g., Temporal Ensembling), which enforces stable predictions under input perturbations or dropout variations through loss terms like mean squared error between multiple predictions (Laine and Aila, 2017); 2) Noise-aware frameworks like DivideMix, which leverage Gaussian Mixture Models (GMM) to separate clean and noisy labels by analyzing loss distributions, achieving robustness in high-noise scenarios (>50% noise) but struggling with low-noise cases due to overlapping distributions (Li et al., 2020b). Advantages include reduced annotation costs and improved generalization through pseudo-labeling unlabeled data, while limitations involve sensitivity to noise thresholds (e.g., GMM failures in <20% noise) and computational complexity from iterative co-training.

Reinforcement learning is a learning paradigm concerned with learning to control a system so as to maximize a numerical performance measure that expresses a long-term objective (Mnih et al., 2015). For example, reinforcement learning could be applied to evaluate treatment policies for patients with hepatitis C virus (Oselio et al., 2022), adjust dynamic treatment regimes in HIV (Yu et al., 2019), push the boundaries of coarse-grained vaccine models (Faris et al., 2022), support outbreak management (Kao et al., 2024), etc. Key technical approaches include value functions (such as Q-learning), policy gradient methods and deep reinforcement learning models (such as DQN). For example, DQN combines empirical playback and target networks to solve stability problems in high-dimensional state Spaces (Mnih et al., 2015). Policy gradient methods (such as PPO) directly optimize policy parameters and are suitable for continuous action Spaces (Schulman et al., 2017). However, the low sample efficiency and exploration-utilization tradeoff of RL remain challenges. Lillicrap et al. proposed depth deterministic strategy gradient (DDPG) for continuous control tasks (Lillicrap et al., 2019). The advantages include the need for prior knowledge and the ability to adapt to dynamic environments, but the disadvantages are significant: high computational cost, long training time, and unpredictable behavior may be generated in complex scenarios, leading to safety and ethical risks (Kulkarni et al., 2016).

## Machine learning in clinical microbiology and infectious diseases

The applications of machine learning in clinical microbiology and infectious diseases include predicting drug targets or vaccine candidates, diagnosing microorganisms causing infectious diseases, classifying drug resistance against antimicrobial medicines, predicting disease outbreaks, and exploring microbial interactions (Goodswen et al., 2021). From the perspective of experienced clinicians, machine learning’s substantial progress carries potential implications across the scope of practice, including diagnosis, risk stratification and prognosis, treatment selection, response prediction, prevention, and control of infectious diseases. The common infectious diseases in clinics mainly include bacterial infection, viral infection, and fungal infection. The Image analysis AI (IAAI) tools are beginning to penetrate routine clinical microbiology practice, and their scope and impact on routine clinical microbiology practice will continue to grow (Burns et al., 2023). The use of machine learning as a means for the discrimination of diseases from mass spectrometric data aims to develop diagnostic and prognostic tools, treatment targets, and patient management systems (Liebal et al., 2020). Representative studies detailing machine learning’s applications in clinical microbiology and infectious diseases are summarized in **Table 1**. As shown in the **Figure 1**, the applications of machine learning in clinical microbiology and infectious diseases include etiology diagnosis, risk stratification, prognosis, treatment selection, treatment innovation, prevention and control.

**Table 1:** Representative machine learning in clinical microbiology and infectious diseases publications.

Study	Application	Method	Results	title
Weinan Dong et al. (Dong et al.., 2024)	Diagnosis	NN	A simplified model for HBV using patients' physical complaints and parameters was developed with good discrimination (AUC = 0.78) and calibration (goodness of fit test p-value >0.05).	Development and validation of HBV surveillance models using big data and machine learning
Yining Bao et al. (Bao et al.., 2021)	Diagnosis	GBM	Gradient boosting machine (GBM) achieved the highest area under the receiver operator characteristic curve for HIV (76.3%) and sexually transmitted infections(syphilis, 85.8%; gonorrhoea, 75.5%; chlamydia, 68.0%).	Predicting the diagnosis of HIV and sexually transmitted infections among men who have sex with men using machine learning approaches
Zhenyu Wei et al. (Wei et al.., 2023)	Diagnosis	LR	The diagnostic model classified the external validation dataset with a sensitivity of 0.907 (0.774, 1.000), specificity 0.899 (0.750, 1.000), accuracy 0.905 (0.805, 1.000), and AUC 0.903 (0.808, 0.998).	Development and multi-center validation of machine learning model for early detection of fungal keratitis
Estela et al. (Lima et al.., 2020)	Diagnosis	RF	The proposed combination of these two analytical methods resulted in the identification of a set of 19 PCM biomarkers that show accuracy of 97.1%, specificity of 100%, and sensitivity of 94.1%.	Metabolomics and Machine Learning Approaches Combined in Pursuit for More Accurate Paracoccidioidomycosis Diagnoses
Mahmoud Essalat et al. (Essalat et al.., 2023)	Diagnosis	CNNs	Densenet161 had the best performance among these models, with an accuracy, precision, recall, and F1 score of 93.55%, 92.52%, 94.77%, and 96.93%, respectively.	Interpretable deep learning for diagnosis of fungal and acanthamoeba keratitis using in vivo confocal microscopy images
Jeany et al. (Delafiori et al.., 2021)	Diagnosis、 Risk stratiffcation	(ADA tree boosting (ADA), gradient tree boosting (GDB)	The best final results were obtained with gradient tree boosting (GDB) to COVID-19 automated diagnosis with 96.0% of specificity and 83.1% of sensitivity. The best results for risk assessment were obtained with ADA Boosting algorithm with 80.3% of specificity and 85.4% of sensitivity, from blind test.	Covid-19 Automated Diagnosis and Risk Assessment through Metabolomics and Machine Learning
Ting Feng et al. (T et al.., 2023)	Risk stratiffcation	Ensemble-based boosted decision trees	Our best performing infection risk model achieves a cross-validated AUC of 0.88 at 1 h before clinical suspicion and maintains an AUC >0.85 for 48 h before suspicion by aggregating information across demographics and a set of 163 vital signs and laboratory measurements.	Machine learning-based clinical decision support for infection risk prediction
Zhuo Wang et al. (Z et al.., 2023)	Risk stratiffcation	Extreme gradient boosting (XGBoost)	To validate the accuracy of our models, we externally tested on an independent cohort and achieved impressive results with an area under the receiver operating characteristic curve of 0. 94, 0.90, 0.86 and 0.91, and an area under the precision-recall curve of 0.93, 0.87, 0.87 and 0.81, respectively, for oxacillin, clindamycin, erythromycin and trimethoprim-sulfamethoxazole.	A risk assessment framework for multidrug-resistant Staphylococcus aureus using machine learning and mass spectrometry technology
Nathaniel et al. (Nj et al.., 2023)	Risk stratiffcation	Classification Tree Analysis (CTA)	The final machine learning model was highly accurate (receiver operating characteristic [ROC] area = 0.775) in training and jackknife validity analyses.	Machine Learning To Stratify Methicillin-Resistant Staphylococcus aureus Risk among Hospitalized Patients with Community-Acquired Pneumonia
Zhiyan Fan et al. (Z et al.., 2023)	Prognosis	XGBoost	According to the areas under the ROC curve (AUC) and DCA results for the training cohort, XGBoost model exhibited excellent performance with F1 Score of 0.847, 0.715, 0.765 and AUC (95% CI) of 0.91 (0.90, 0.92), 0.78 (0.76, 0.80), and 0.83 (0.81, 0.85) in 7 days, 14 days and 28 days group, respectively.	Construction and validation of prognostic models in critically Ill patients with sepsis-associated acute kidney injury: interpretable machine learning approach
Junyu Liu et al. (Liu et al.., 2023)	Prognosis	LR	An artificial intelligence (AI) model was trained to detect and count cryptococci, and the mean average precision (mAP) was 0.993.	Development and validation of a machine learning model to predict prognosis in HIV-negative cryptococcal meningitis patients: a multicenter study
Alexandre et al. (de Fátima Cobre et al., 2022)	Prognosis	PLS-DA	The PLS-DA model presented the best performance for both datasets, with accuracy rates to predict the diagnosis, severity and fatality of COVID-19 of 93%, 94% and 97%, respectively.	Diagnosis and prognosis of COVID-19 employing analysis of patients' plasma and serum via LC-MS and machine learning
Mathew et al. (Stracy et al.., 2022)	Prevention	LR	The models predict the risk of resistance emergence well (the area under the curve ranged from 0.89 for nitrofurantoin to 0.62 for amoxicillin/CA in UTIs, and from 0.96 for amoxicillin/CA to 0.58 for cefuroxime in wound infections.	Minimizing treatment-induced emergence of antibiotic resistance in bacterial infections
Andrew et al. (Gao et al.., 2022)	Treatment selection	RF	The model attained 96.72% accuracy for classifying between active and inactive drug compounds.Several drugs, including goserelin and icatibant, were detected as active with high confidence.	Machine-learning-based virtual screening to repurpose drugs for treatment of Candida albicans infection
N M Smith et al. (Smith et al.., 2020)	Treatment selection	Genetic algorithm(GA)	A mechanism-based model of the data and population pharmacokinetics of each drug were used to develop a GA to define the optimal regimen parameters.Monotherapies resulted in regrowth to ~1010cfu/mL by 24 h, while combination regimens employing high-intensity polymyxin B (PMB) exposure achieved complete bacterial eradication (0 cfu/mL) by 336 h.	Using machine learning to optimize antibiotic combinations: dosing strategies for meropenem and polymyxin B against carbapenem-resistant Acinetobacter baumannii
Felix et al. (Wong et al.., 2024)	Treatment innovation	NN	Of these structural classes of compounds, one is selective against methicillin-resistant S. aureus (MRSA) and vancomycin-resistant enterococci, evades substantial resistance, and reduces bacterial titres in mouse models of MRSA skin and systemic thigh infection.	Discovery of a structural class of antibiotics with explainable deep learning
Célio et al. (Santos-Júnior et al.., 2024)	Treatment innovation	Macrel—(Meta)genomic AMP Classification and Retrieval system	To validate our predictions, we synthesized and tested 100 AMPs against clinically relevant drug-resistant pathogens and human gut commensals both in vitro and in vivo. A total of 79 peptides were active, with 63 targeting pathogens.	Discovery of antimicrobial peptides in the global microbiome with machine learning
Jennifer et al. (Dawkins et al.., 2022)	Muti-omics	LR, RF	Using predictive statistical/machine learning models, we demonstrated that the metabolomic data, but not the other data sources, can accurately predict future recurrence at 1 week (AUC 0.77 [0.71, 0.86; 95% interval]) and 2 weeks (AUC 0.77 [0.69, 0.85; 95% interval]) post-treatment for primary CDI.	Gut metabolites predict Clostridioides difficile recurrence
Jing Cao et al. (Cao et al.., 2023)	Muti-omics		A classifier (DPFs-DL) for viral versus bacterial infection discrimination (AUC of 0.775) and coronavirus disease 2019 (COVID-2019) diagnosis (AUC of 0.917) is also built. Furthermore, a metabolic biomarker panel of two differentially regulated metabolites, which may serve as potential biomarkers for COVID-19 management (AUC of 0.677-0.883), is constructed.	Deep Learning of Dual Plasma Fingerprints for High-Performance Infection Classification

### Etiology diagnosis

In clinical microbiology and infectious diseases, machine learning shows promise and practicability, both in doing existing tasks and making broader applications of existing data than traditional diagnosis does. As detailed in a review by Stephen and colleagues, previous approaches to processing microbiological data entailed identifying and sequencing pathogenic microorganisms, algorithmically extracting features from them, and using those features for classification (Goodswen et al., 2021).

Many studies have reported the practical application of machine learning in the diagnosis of bacterial infections. Rare event detection by machine learning can be used for screening purposes or final identification of a microbe including microscopic detection of mycobacteria in a primary specimen, detection of bacterial colonies growing on nutrient agar, or detection of parasites in a stool preparation or blood smear (Burns et al., 2023). Score-based image analysis AI can be applied to a scoring system that classifies images in toto as its output interpretation and examples include application of the Nugent score for diagnosing bacterial vaginosis and interpretation of urine cultures (Burns et al., 2023). Leveraging machine learning can distinguish between bacterial and viral-induced pharyngitis using hematological markers (Jin et al., 2023). Profiling of the conjunctival bacterial microbiota reveals the feasibility of utilizing a microbiome-based machine learning model to differentially diagnose microbial keratitis and the core components of the conjunctival bacterial interaction network (Ren et al., 2022).

Many studies have reported the practical application of machine learning in the diagnosis of viral infections. Respiratory viruses can be rapidly and quantitatively detected by using surface-enhanced Raman spectroscopy and machine learning (Yang et al., 2022). Image-based and machine learning-guided multiplexed serology test is developed for SARS-CoV-2 diagnosis (Pietiäinen et al., 2023). Blood tests and machine learning can predict the diagnosis of SARS-CoV-2 by calculating the gravity of each feature, such as eosinophils, monocytes, leukocytes, and platelets (Chadaga et al., 2022). Hepatitis B virus(HBV) detection models are developed and validated through a neural network algorithm by using routine clinical data to improve the detection of HBV (Dong et al., 2024). Gradient boosting machine(GBM) using clinical records can predict the diagnosis of HIV and sexually transmitted infections among men who have sex with men using (Bao et al., 2021). PCA-SVM (poly-5) model is effective and robust for clinical prediction of DENV infection in human blood sera (Saleem et al., 2022).

Many studies have reported the practical application of machine learning in the diagnosis of fungal infections. The binary logistic regression model is conducted for early detection of fungal keratitis by learning twelve clinical signs of slit-lamp images and collinear variables (Wei et al., 2023). Metabolomics and machine learning approaches are combined in pursuit of more accurate Paracoccidioidomycosis diagnoses (Lima et al., 2020). Interpretable deep learning can diagnose fungal and acanthamoeba keratitis using *in vivo* confocal microscopy images (Essalat et al., 2023).

### Risk stratification and prognosis

Accurate prediction of risk stratification and prognosis is crucial for balancing the upsides of therapy and the risk of side effects. Although there are still many challenges in specific clinical practice, machine learning provides a reliable way to create efficient models for estimating risk and prognosis.

Machine learning-based clinical decision support is effective for infection risk prediction (Feng et al., 2023). Development and validation of a machine learning-driven prediction model is applied for serious bacterial infections, such as bacterial meningitis or sepsis, among febrile children in emergency departments (Lee et al., 2022). Machine learning can be used for the prediction of prognostic risk factors in patients with invasive candidiasis infection and bacterial bloodstream infection (Li et al., 2022). Virulence factors (VFs), which are crucial for pathogens to successfully infect host tissue and evade the immune system, can be predicted by using sequence alignment percentage and ensemble learning models (Singh et al., 2024). Machine learning can stratify methicillin-resistant Staphylococcus aureus risk among hospitalized patients with community-acquired pneumonia (Rhodes et al., 2023). COVID-19 automated risk assessment uses an ADA tree boosting algorithm through metabolomics data from mass spectrometry (Delafiori et al., 2021). A risk assessment framework for multidrug-resistant Staphylococcus aureus shows high accuracy by incorporating machine learning and mass spectrometry technology (Wang et al., 2023).

The prognosis of infectious diseases is an important basis for clinical adjustment of treatment and machine learning provides important and efficient tools for infection prognosis. A machine learning model for predicting prognosis in HIV-negative CM patients was built and validated, and the model might provide a reference for personalized treatment of HIV-negative CM patients (Liu et al., 2023). Prognostic models in critically Ill patients with sepsis-associated acute kidney injury can be constructed and validated through an interpretable machine-learning approach (Fan et al., 2023). Prediction of prognosis in elderly patients with sepsis can be based on a random survival forest model (Zhang et al., 2022). A simplified machine learning model utilizes platelet-related genes for predicting poor prognosis in sepsis (Diao et al., 2023). Prognosis in COVID-19 patients can be predicted by using machine learning and readily available clinical data (de Fátima Cobre et al., 2022; Campbell et al., 2021).

### Treatment selection and innovation

Infectious diseases need to take corresponding drug treatment according to the specific cause, such as viral infection, bacterial infection, and fungal infection. Therefore, accurate diagnosis is a prerequisite for scientific selection of treatment plans and examples of accurate diagnosis using machine learning have been described in detail above. This section will focus on the contents of machine learning in treatment selection and innovation.

The choice of treatment for infectious diseases depends first on accurate diagnosis, and then critically on the strategies for drug selection. The problem of antibiotic resistance varies with time, environment, and region, and there are some differences between *in vitro* and *in vivo* results of drug susceptibility tests. Hence, the application of machine learning is an effective tool to solve the above problems. Machine learning can be used for microbial identification and antimicrobial susceptibility testing on MALDI-TOF mass spectra (Weis et al., 2020). Machine-learning-based virtual screening can repurpose drugs for the treatment of Candida albicans infection (Gao et al., 2022). In addition, AI is gaining more and more attention for drug combination discovery and optimization against a variety of infectious agents in bacteria, viruses, parasites, and fungi. In general, input variables that have been used by an AI system for drug combination design can be divided into three groups, such as drug-based, pathogen-based, and host-based (He et al., 2021). Regarding bacteria, three- and four-drug combinations highly efficacious for treating MDR and extensively drug-resistant TB have been identified with the aid of an output-driven feedback system (Silva et al., 2016). In addition, by using genetic algorithms, the dosing strategies of meropenem/polymyxin B combination against carbapenem-resistant A. baumannii were optimized (Smith et al., 2020). Concerning viruses, AI platforms have been developed to discover the optimal combination therapies for HIV, HBV, hepatitis C virus (HCV), SARS-CoV-2, Ebola, vesicular stomatitis virus, herpes simplex virus-1, using a series of machine learning models, such as decision trees, SVM, Bayesian network, logistic regression, Random forest (He et al., 2021; Churkin et al., 2022; Bukic et al., 2023). For fungi, a novel computational algorithm termed Network-based Laplacian regularized Least Square Synergistic drug combination prediction has been developed to predict synergistic drug combinations for fungal diseases where drug resistance is common (Chen et al., 2016).

The antibiotic resistance crisis is a major challenge facing humanity today, and machine learning is one of the effective tools to address it. A machine-learning-based approach is presented to predict active antimicrobial peptides (AMPs) within the global microbiome and leverage a vast dataset of 63,410 metagenomes and 87,920 prokaryotic genomes from environmental and host-associated habitats to create the AMPSphere, a comprehensive catalog comprising 863,498 non-redundant peptides (Santos-Júnior et al., 2024). Using explainable graph algorithms, substructure-based rationales are identified for compounds with high predicted antibiotic activity and low predicted cytotoxicity, and after testing of 283 compounds, it is assumed that one is selective against methicillin-resistant S. aureus (MRSA) and vancomycin-resistant enterococci, evades substantial resistance, and reduces bacterial titers in mouse models of MRSA skin and systemic thigh infection (Wong et al., 2024). Leveraging machine learning essentiality predictions and chemogenomic interactions to identify the glutaminyl-tRNA synthetase Gln4 as the antifungal targets of N-pyrimidinyl-β-thiophenylacrylamide (NP-BTA) (Fu et al., 2021). In addition, the high variability of the virus is a difficulty in the development of antiviral drugs. With the integrated efforts to improve data quality and availability, ML is a promising approach to developing next-generation antivirals and therapeutics for infectious diseases (Kumari et al., 2023). For instance, ML methods can design small molecules based on multiscale behavior and interactions to selectively inhibit multiple influenza targets while mitigating interaction with host proteins to minimize adverse effects (Overhoff et al., 2021).

### Prevention and control

Preventing and controlling infectious diseases remains a global public health challenge, as it sometimes causes unexpected pandemics, which are responsible for high morbidity, mortality, and substantial economic impact. AI has had a pivotal role in the prevention and control of infectious diseases. AI has shown great potential in developing effective HIV prevention intervention strategies (Xiang et al., 2022). Machine learning methods can predict the epidemic of human-adaptive Influenza A Viruses based on viral nucleotide compositions (Li et al., 2020a). As most infections are seeded from a patient’s microbiota, these resistance-gaining recurrences can be predicted using the patient’s past infection history and minimized by machine learning-personalized antibiotic recommendations, offering a means to reduce the emergence and spread of resistant pathogens (Stracy et al., 2022). Furthermore, the DT models with alternative sensitivity levels can be exploited in different stages of an emerging infectious diseases(EID) disaster to optimize medical resource allocation, which is crucial in the response to a large-scale epidemic of emerging infectious disease (Chiu et al., 2022).

Vaccines are automatic immune preparations made by artificially attenuated, inactivated, or genetically modified pathogenic microorganisms (such as bacteria, rickettsia, viruses, etc.) and their metabolites for the prevention of infectious diseases. Few developments have done more to limit the spread of infectious disease and associated mortality than the advent of vaccination (Dubé et al., 2021). Vaxign2 is updated to the second generation of the first Web-based vaccine design program using reverse vaccinology and machine learning (Ong et al., 2021). The newly developed machine learning-based reverse vaccinology tools are applied to design the COVID-19 vaccine (Ong et al., 2020; Lv et al., 2021; Huffman et al., 2022). A random forest model is used for active vaccine safety monitoring, such as anaphylaxis and agranulocytosis (Kim et al., 2021). A combinatorial artificial-neural-network design-of-experiment (ANN-DOE) model shows great advantages in lipid nanoparticle-based mRNA vaccine bioprocess (Maharjan et al., 2023). Supervised and unsupervised machine learning approaches are used for monitoring subvisible particles within an aluminum-salt adjuvanted vaccine formulation (Greenblott et al., 2024).

### Machine learning in multi-omic data

With the development of technologies such as next-generation DNA and RNA sequencing, it becomes more feasible to obtain personalized data about complex diseases. Data from various omics sources such as genomics, proteomics, metabolomics, transcriptomics, lipidomics, immunomics, glycomics, radiomics (Priya et al., 2022), and ultrasonics can be integrated to unravel the intricate working of systems biology using machine learning-based predictive algorithms (Reel et al., 2021). Integrating multi-omics data with electronic health records (EHRs) can be used for precision medicine by using advanced artificial intelligence (Tong et al., 2024). Integrating multi-omics data could reveal the host-microbiota interactome in inflammatory bowel disease (Su et al., 2025), the interplay between gut microbiome and the host following opioid use (Kolli et al., 2023), host responses to lethal human virus infections (Eisfeld et al., 2024) and etc. HONMF, which is the AI system for the integrative analysis of multi-modal microbiome data, including bacterial, fungal, and viral composition profiles, provides rich biological insights by implementing discriminative microbial feature selection and bacterium-fungus-virus association analysis (Ma et al., 2023). On the COVID-19 diagnosis task, omics-based models performed better than image or physiological feature-based models, proving the importance of the omics-based dataset for future model development (Liu X. et al., 2023). A comprehensive multi-omic blood atlas is presented for identifying immune signatures and correlates of host response with varying COVID-19 severity in an integrated comparison with influenza and sepsis patients versus healthy volunteers (David et al., 2022). The muti-omic machine learning model has implications for the development of diagnostic tests and treatments that could ultimately short-circuit the cycle of Clostridioides difficile infection (CDI) recurrence, by providing candidate metabolic biomarkers for diagnostics development, as well as offering insights into the complex microbial and metabolic alterations that are protective or permissive for recurrence (Dawkins et al., 2022). Deep learning of dual plasma fingerprints is developed for high-performance infection classification (Cao et al., 2023). Using lasso and sparse CCA to detect specific associations between gut microbial taxa and host genes, the study finds that Peptostreptococcaceae is associated with MAPK3 and VIPR1 that are part of G protein-coupled receptors pathways in inflammatory bowel disease; and *Bacteroides massiliensis* is associated with the host gene PLA2G4A, a member of the prostaglandin biosynthesis pathway, in irritable bowel syndrome (Priya et al., 2022).

## The key to machine learning for clinicians

Professional training in statistical and research methods has long been a cornerstone of medical education for clinicians. However, it is unrealistic and unnecessary for clinicians to completely understand machine learning’s complexity and depth at the level of a computer scientist. Owing to the diversity and complexity of data types encountered, microbiological data often require individualized solutions for dealing with them effectively, and this makes it difficult to recommend common tools or guidelines for the application of machine learning in these specific domains, as the model selection, training procedure, and test data will reply highly on the exact questions one wants to answer. Clinicians need to master the basic concepts, core steps, general limitations, and common applications of machine learning, such as data processing, feature selection and extraction, model selection and evaluation, generalization, overfitting, underfitting, etc.

### Data processing

When available data are in larger quantities, clinicians need to consider more highly parameterized models such as deep neural networks. In supervised machine learning, the relative proportions of each ground truth label in the dataset should also be considered, with more data required for machine learning to work if some labels are rare (Wei and Dunbrack, 2013). Considering the data leakage, clinicians have to pay attention to the problem of having related samples in the training and testing sets. It is usually necessary to use 70-80% of the total data set as the training set and 20-30% as the independent test set (Collins and Moons, 2019). The’related’ here depends on the nature of the study, which might be a case of sampling data from the same patient or the same organism. The issue of data leakage becomes a problem when a model that appears accurate on some benchmark set performs poorly on new data that are different from the training set; in other words, the model does not generalize, likely because it has not modeled the true relationship between the variables, but rather remembered hidden associations present in the data (Greener et al., 2022). Strategies to prevent data leakage include proper data splitting (Kaufman et al., 2012), pipelines for preprocessing (Fabian et al., 2011), time-aware validation (Bergmeir and Benítez, 2012), causal feature analysis (Pearl, 2009), potential leak characteristics identification (Lundberg and Lee, 2017) and so on.

### Feature selection and extraction

The main goal of feature selection is the minimization of the original amount of input features, which is chosen for training the machine learning model. It is different from feature extraction, which refers to generating new features from a large number of input features. The ‘omics’ technologies used for microbiome analysis continuously evolve and, although much of the research is still at an early stage, large-scale datasets of ever-increasing size and complexity are being produced (Cammarota et al., 2020). Facing the high-dimensional data, both feature selection and feature extraction can generalize and simplify the input features of the machine learning model.

There are some tips for feature selection and extraction techniques. Firstly, the application of any learning tools for evaluating prediction performance can promote the iterative removal or addition of features to identify those that seem redundant or provide no new information. Secondly, some machine learning algorithms already contain feature selection steps, for example, SVMs embed recursive feature elimination, RF provides a feature importance score, and the LASSO constrains most regression coefficients to be exactly zero. Thirdly, dimensionality reduction is fairly effective for feature extraction when extreme reduction of the high-dimension data is needed and is unnecessary to retain the original features within the model.

### Model selection and evaluation

Clinicians usually focus on accurate modeling, discovering mechanisms and the factors responsible for modeling output. The step of model selection exploits the training data to identify the best machine learning model based on the evaluation of different types of models, or across models of the same type but with different hyperparameter settings (Asnicar et al., 2024). In machine learning, the commonly used model evaluation metrics include accuracy, precision, recall, F1 score, receiver operating characteristic (ROC) curve, AUC, mean squared error(MSE), mean absolute error(MAE), log loss, R-squared, cross-validation score, etc (Rainio et al., 2024). Accuracy is the fraction of correct predictions overall predictions. Precision is the fraction of true positives overall positives. Recall or sensitivity is the fraction of true positives over all correct predictions. Specificity is the fraction of true negatives over all negatives. The F1 score is the harmonic mean of precision and recall. ROC curve plots pairs of specificity and sensitivity values calculated at all possible threshold scores. The area under the ROC curve (AUC-ROC) summarizes the performances regardless of the threshold and ranges from 0.5 (random classification) to 1.0 (perfect classification). MSE is essentially finding the average squared error between the predicted value and the true value. MAE is the average of all absolute errors, which finds the average absolute distance between the predicted value and the true value. Log loss is mainly used in binary classification problems to measure the difference between the predicted results of the model and the real label. R (Cornet, 2013), also known as the coefficient of determination, represents how well the model fits the data. An R (Cornet, 2013) representation model close to 1.0 agrees well with the data, while a model close to 0 does not. The cross-validation score evaluates the performance of the model on the new data set by dividing the data set into a training set and a test set to prevent overfitting and improve the generalization ability of the model.

As a data science professional, it is essential to understand the above important evaluation metrics. Clinicians need to understand their uses, advantages, and disadvantages, which will help you choose and implement them accordingly. Classification is one of the most widely used problems in machine learning, with various industrial applications, such as face recognition, image classification, content review, text classification, etc. SVM, LR, DC, RF, and other models are also some of the most popular classification models. The most commonly used metrics for classification problems are accuracy, precision, recall, F1 score, ROC curve and AUC, log loss, etc. In addition, the commonly used metrics for multi-label problems in classification are mainly precision at k (P@k), average precision at k (AP@k), mean average precision at k (MAP@k), etc. Regression models are used to predict continuous target values and also have a wide range of applications, such as house price forecasting, weather forecasting, stock price forecasting, etc. LR, RF, XGboost, RNN, etc., are also some of the most popular regression models. The most common metrics in regression are MAE, MSE, Root mean squared error (RMSE), Root mean squared logarithmic error (RMSLE), Mean percentage error (MPE), Mean absolute percentage error (MAPE), R (Cornet, 2013), etc. Choosing appropriate strategies to evaluate machine learning models is important to provide robust and generalizable estimations and avoid biased models (Topçuoğlu et al., 2020).

## Conclusion

With the popularization and development of machine learning technology, the medical field has also undergone subversive changes and challenges. In the field of clinical microbiology and infectious diseases, machine learning has greatly promoted the diversification and accuracy of diagnostic methods, scientific decision-making of treatment programs, accurate judgment of disease prognosis, innovation of treatment means, and effective prevention of diseases. While many of these applications are at the exploratory stage and require further validation and generalization, they hold substantial promise in furthering clinical practice. Different algorithms of machine learning have their own advantages and disadvantages, which should be comprehensively judged and used in combination with the actual situation.

Clinical microbiologists and infectiologists are deeply immersed in the fields of data science and artificial intelligence, by focusing on the general principles and guidelines and on avoiding frequent potential issues affecting machine learning ranging from evaluation issues to study design problems. Choosing the right machine learning algorithm and scientific evaluation model is vitally important because it can help to generalize the model and avoid the problems of underfitting and overfitting. In clinical microbiology and infectious diseases, a large number of multi-omics data is a problem we have to face, which is also an important direction to guide our future research and development.
